# Dietary normalization from a fat, fructose and cholesterol-rich diet to chow limits the amount of myocardial collagen in a Göttingen Minipig model of obesity

**DOI:** 10.1186/s12986-018-0303-x

**Published:** 2018-09-25

**Authors:** Laura Jul Andreasen, Simone Krog, Trine Pagh Ludvigsen, Ole Lerberg Nielsen, Jacob Eifer Møller, Berit Østergaard Christoffersen, Henrik Duelund Pedersen, Lisbeth Høier Olsen

**Affiliations:** 1 Department of Veterinary and Animal Sciences, Faculty of Health and Medical Sciences, University of Copenhagen, Ridebanevej 9, 1870 Frederiksberg, Denmark; 2 Global Drug Discovery, Novo Nordisk A/S, Novo Nordisk Park 1, 2760 Måløv, Denmark; 3 Department of Cardiology, Odense University Hospital, Sdr Boulevard 29, 5000 Odense C, Denmark; 4 Ellegaard Göttingen Minipigs, Soroe Landevej 302, 4261 Dalmose, Denmark

**Keywords:** Dietary intervention, Obesity, Porcine model, Left ventricular remodeling, Collagen, Hydroxyproline, Cardiomyocyte hypertrophy

## Abstract

**Background:**

Dietary interventions have been shown to attenuate some of the myocardial pathological alterations associated with obesity. This study evaluated the effect of dietary normalization from a fat/fructose/cholesterol-rich diet to chow on left ventricular (LV) myocardial fibrosis, fat infiltration and hypertrophy but also the specific influence of obesity, plasma lipids and glucose metabolism markers on heart morphology in a Göttingen Minipig model of obesity.

**Methods:**

Forty castrated male Göttingen Minipigs were assigned to three groups fed either standard minipig chow (SD, *n* = 8) for 13 months, fat/fructose/cholesterol-rich diet (FFC, *n* = 16) for 13 months or fat/fructose/cholesterol-rich diet for 7 months and then changed to standard minipig chow for the remaining 6 months (FFC/SD, *n* = 16). Body weight, body fat percentage, plasma lipids and glucose metabolism markers were evaluated in all three groups after 6–7 months (prior to diet adjustment for FFC/SD) and again before termination. Further, biochemical quantification of myocardial collagen and triglyceride content, semi-quantitative histological evaluation of fibrosis and fat infiltration and quantitative histological analysis of collagen and cardiomyocyte diameter were performed and heart weight was obtained after termination. Group differences were evaluated using Kruskal-Wallis test and Fisher’s exact test for categorical variables. Pearson correlation analysis was performed to test for correlations between myocardial changes and selected explanatory variables. For non-parametric response variables, a Spearman correlation analysis was applied.

**Results:**

Myocardial collagen content quantified biochemically was significantly lower in FFC/SD compared to FFC (*P* = 0.02). Furthermore, dietary normalization from a fat/fructose/cholesterol-rich diet to chow caused stagnation of body weight and body fat percentage, normalized intravenous glucose tolerance index (K_G_) and plasma lipid levels.

**Conclusion:**

Dietary normalization led to lower LV collagen content in obese Göttingen Minipigs. Despite gross obesity and significant deteriorations in glucose and lipid metabolism, only mild myocardial changes were found in this model of obesity and therefore further model optimization is warranted in order to induce more severe myocardial changes before dietary or pharmacological interventions.

## Background

Obesity is a steadily increasing health issue, and the worldwide prevalence of obesity has nearly tripled between 1975 and 2016 [1]. This is a major concern because obesity is associated with an increased risk of cardiovascular disease [2, 3], including heart failure (HF) [2, 4]. Specifically, obesity is associated with myocardial accumulation of intra-myocellular lipids causing lipoapoptosis [5–7], left ventricular (LV) hypertrophy [8, 9], and to some degree increased myocardial fibrosis [10, 11]. These obesity-induced myocardial alterations may contribute to impairment of LV function [7, 12–15] and ultimately HF.

Dietary interventions accompanied by weight loss have been shown to reduce both LV hypertrophy and myocardial triglyceride content in obese humans [16–19]. Not much is known about the impact of dietary change on myocardial fibrosis in humans, but studies in obese rodents have shown that weight loss obtained by calorie restriction can attenuate myocardial fibrosis [20, 21]. Besides weight loss and reversal of myocardial remodeling, dietary interventions often reduce dyslipidemia and systemic insulin resistance [16, 17, 19, 22]. Improvements in insulin resistance without weight loss have been associated with reversal of LV function abnormalities [22], stating a possible primary effect of insulin resistance. However, the underlying mechanisms of the attenuation of myocardial remodeling are not yet fully understood. Translational animal models mimicking human obesity could facilitate more effective research in this field, enabling investigations of the underlying mechanisms and providing a better understanding of the related co-morbidities.

Over the past 30 years the use of porcine models in cardiovascular research has grown [23]. Compared to rodents, the pig is more similar to humans in terms of lifespan, anatomy, physiology, and metabolism [24]. More importantly, when investigating obesity, the pig displays many of the same metabolic abnormalities as humans when fed excess calories [25–27]. Additionally, myocardial fibrosis, fat infiltration and cardiac hypertrophy have been described in different breeds of obese pigs [27–30].

Using a fat/fructose/cholesterol-rich diet-induced minipig model of obesity, the aim of this study was to test the hypotheses that dietary normalization from a fat/fructose/cholesterol-rich diet to chow would 1) limit the development of LV myocardial fibrosis, fat infiltration, and hypertrophy and 2) improve plasma lipids and glucose metabolism markers. Furthermore, we hypothesized that 3) overall, the degree of obesity and level of plasma lipids and glucose metabolism markers would be associated with heart morphology.

## Methods

This study is part of a large prospective intervention study in Göttingen Minipigs investigating diet-induced atherosclerosis and diabetes- and obesity-related abnormalities in the heart, liver and kidneys. Therefore some of the presented groups and background characteristics will also be published elsewhere in other contexts. The study was approved by the Animal Experimental Inspectorate, Ministry of Justice, Denmark.

### Animals, experimental diets and feeding

Castrated male Göttingen Minipigs aged 6-7 months (Ellegaard Göttingen Minipigs A/S, Dalmose, Denmark) were housed at the Experimental Animal Unit at University of Copenhagen for the 13 months duration of study. Animals were single-housed during periods with central venous access (intravenous (IV) catheters) and otherwise group-housed. Animals had free access to bedding material and water, and were kept at a natural day/night cycle.

**Fig. 1:**
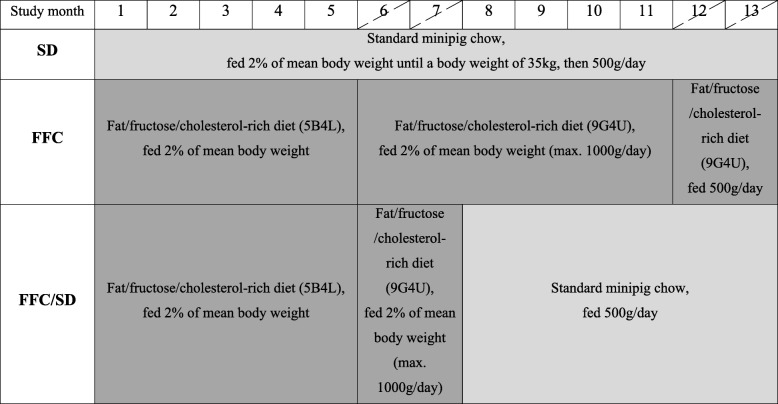
Overview of study design with focus on diet paradigm. Hatched study months indicate the two in vivo assessment periods: mid-study and before termination. Approximate timing common for all three cohorts. 5B4L and 9G4U indicate the two different fat/fructose/cholesterol-rich diets used. Nutrient profile differences between the two diets can be found in the text. SD = fed standard chow, FFC = fed fat/fructose/cholesterol-rich diet, FFC/SD = dietary normalization

Overview of the study design and feeding paradigm can be seen on Fig. 1. Study design was staggered with a 5-week interval between three cohorts of animals, and all three study groups were represented in each cohort. Animals were assigned to three different study groups; a lean control group (SD, *n* = 8) fed standard minipig chow that contained 2.8 kcal/g with (percentage by weight) 13.0% protein, 2.1% fats and 52.4% carbohydrate (Mini pig, Special Diet Services, Essex, United Kingdom); an obese group (FFC, *n* = 16) fed fat/fructose/cholesterol-rich diet (9G4U and 5B4L, TestDiets®, Missouri, USA) and a diet normalization group (FFC/SD, *n* = 16) fed fat/fructose/cholesterol-rich diet for 7 months and then changed to standard minipig chow for the remainder of the 13 months study period.

Two different diets with high fat, fructose and cholesterol content were used in the study. 5B4L was used for the first 5 months of the study period [26], whereas, 9G4U was used for the remainder of the study in order to avoid extremely high plasma cholesterol. Both diets contained 4.1 kcal/g with (percentage by weight) 16.7% protein, 19.8% fats and 42.3% carbohydrate. The two fat/fructose/cholesterol-rich diets differed in fructose and cholesterol content, where 5B4L contained (percentage by weight) 2% cholesterol and 17.8% fructose and 9G4U 1% cholesterol and 18.8% fructose.

All animals were fed individually once daily. Animals receiving the fat/fructose/cholesterol-rich diet were fed 2% of body weight (BW) with maximum 1000 g/day. However, 11 months after study initiation animals in FFC were fed more restrictedly with a maximum of 500 g/day due to severe obesity and lameness. Lean control animals were fed according to the breeder’s recommendations (Fig. 1). When animals in FFC/SD were changed to standard minipig chow they were fed 500 g/day.

### In vivo evaluations

In vivo evaluations included intravenous glucose tolerance test (IVGTT), blood sampling and a dual-energy x-ray absorptiometry (DEXA) scan and were all done in overnight fasted animals (approx. 18 h). The evaluations were performed in all three groups of pigs at two time points, 1) mid-study, prior to diet adjustment for FFC/SD and 2) before euthanasia.

An IVGTT was performed 3 to 7 weeks prior to diet adjustment and euthanasia. The test was performed in awake and unrestrained animals using pre-implanted IV catheters in the jugular or cranial caval vein. After baseline samples, an IV bolus of 0.3 g/kg glucose (500 g/L glucose, SAD, Denmark) was given and blood was sampled at 2, 5, 10, 15, 20, 30, 40, 50, 60 and 90 min after the bolus. Blood was EDTA-stabilized and kept on wet ice maximum 30 min before centrifugation at 2000×g for 10 min at 4 °C. Plasma was stored at − 80 °C until further analysis, except plasma used for glucose analysis which was performed on the day of IVGTT. Intravenous glucose tolerance index (K_G_) was calculated as the negative slope of the linear regression of the natural logarithm to glucose vs. time from 5 to 30 min of IVGTT and area under the curve for insulin response was calculated at sampling time 0–60 min (AUC_Insulin_), both as previously described [26, 31]. Blood samples for evaluation of plasma triglyceride and total cholesterol levels were obtained 2-3 days prior to IVGTT, and handled as described above.

Body weight was assessed on a weekly basis. One to 2 weeks prior to diet adjustment and euthanasia a full-body scan using DEXA (GE Lunar Prodigy, GE Healthcare, Brøndby, Denmark) was performed in anaesthetized animals to assess body fat percentage and lean body mass (LBM). Relative heart weight was calculated as the ratio of heart weight to LBM, and as the ratio of heart weight to BW.

### Plasma analyses

Plasma glucose was analyzed using Biosen auto analyzer (BIOSEN, S_Line, EKF Diagnostics, Cardiff, United Kingdom) according to the manufacturer’s instructions. Plasma samples were also analyzed for porcine insulin content using Luminescent Oxygen Channeling Immunoassay as described elsewhere [32] with few modifications regarding antibodies (biotinylated mAb OXI005 was used instead of bioinylated X14-6F34). Both triglyceride and total cholesterol concentrations were analyzed using Cobas® 6000 auto analyzer (Roche, Diagnostics Limited, Rotkreuz, Switzerland) according to the manufacturer’s instruction. High-performance liquid chromatography using Superose 6 columns (10/300, GE Healthcare, Brøndby, DK) in an automatized system (Agilent 1100 HPLC system, Agilent Technologies, Waldbronn, GE) was used to estimate group cholesterol fractions; high-density lipoprotein cholesterol (HDL-C), low-density lipoprotein cholesterol (LDL-C), very-low-density lipoprotein cholesterol (VLDL-C). Pooled plasma samples from termination were used and handled as previously described [26]. In short, two to three animals with total plasma cholesterol concentrations close to the group mean were selected (SD *n* = 2, FFC *n* = 3 and FFC/SD *n* = 3). Effort was made to select animals so that each cohort was represented.

### Euthanasia and tissue preparation

Animals were euthanized in surgical anesthesia by bleeding from the axillae. Hereafter the heart was removed from the animals and weighed. For biochemical analyses 2x2x11mm transmyocardial samples from LV free midwall were snap-frozen in liquid nitrogen and stored at − 80 °C until use. Transmyocardial samples from LV free midwall were also collected for histological analyses. Samples were fixated in 4% neutral buffered formaldehyde for 28 h ± 5 h, dehydrated, and then embedded in paraffin wax. Two tissue sections of 4 μm were either stained with hematoxylin and eosin (H&E) or picro sirius red (PSR) with Weigert’s iron hematoxylin nuclear staining. All sections were digitalized using a NanoZoomer XR (Hamamatsu Photonics, Japan) scanner. Sections with PSR staining were scanned at 20X and H&E sections at 40X magnification.

### Histological analyses

#### Semi-quantitative analysis of LV myocardial fat infiltration and fibrosis

Degree of LV myocardial interstitial, epicardial and perivascular fat infiltration was scored manually on digitalized H&E stained sections using a scale 0/1+/2++/3+++, modified from van Hoeven & Factor [33]. In addition, the degree of interstitial, replacement and perivascular fibrosis was scored on PSR stained sections. Vessel average scores of perivascular fat infiltration and perivascular fibrosis were calculated for each animal. The observer was blinded for group allocation in all histological analyses.

#### Quantitative analysis of LV myocardial collagen

Digital image analysis software Biotopix™ for Windows 7, version 7.0.0.2899 (Visiopharm®, Hørsholm, Denmark) was used to quantify LV myocardial collagen fractions on PSR stained sections. Algorithms were designed to detect collagen and remaining myocardial tissue and create regions of interest excluding epi- and endocardium. Manual corrections were performed if needed. A collagen fraction area was calculated.

#### Left ventricular cardiomyocyte size

**Fig. 2:**
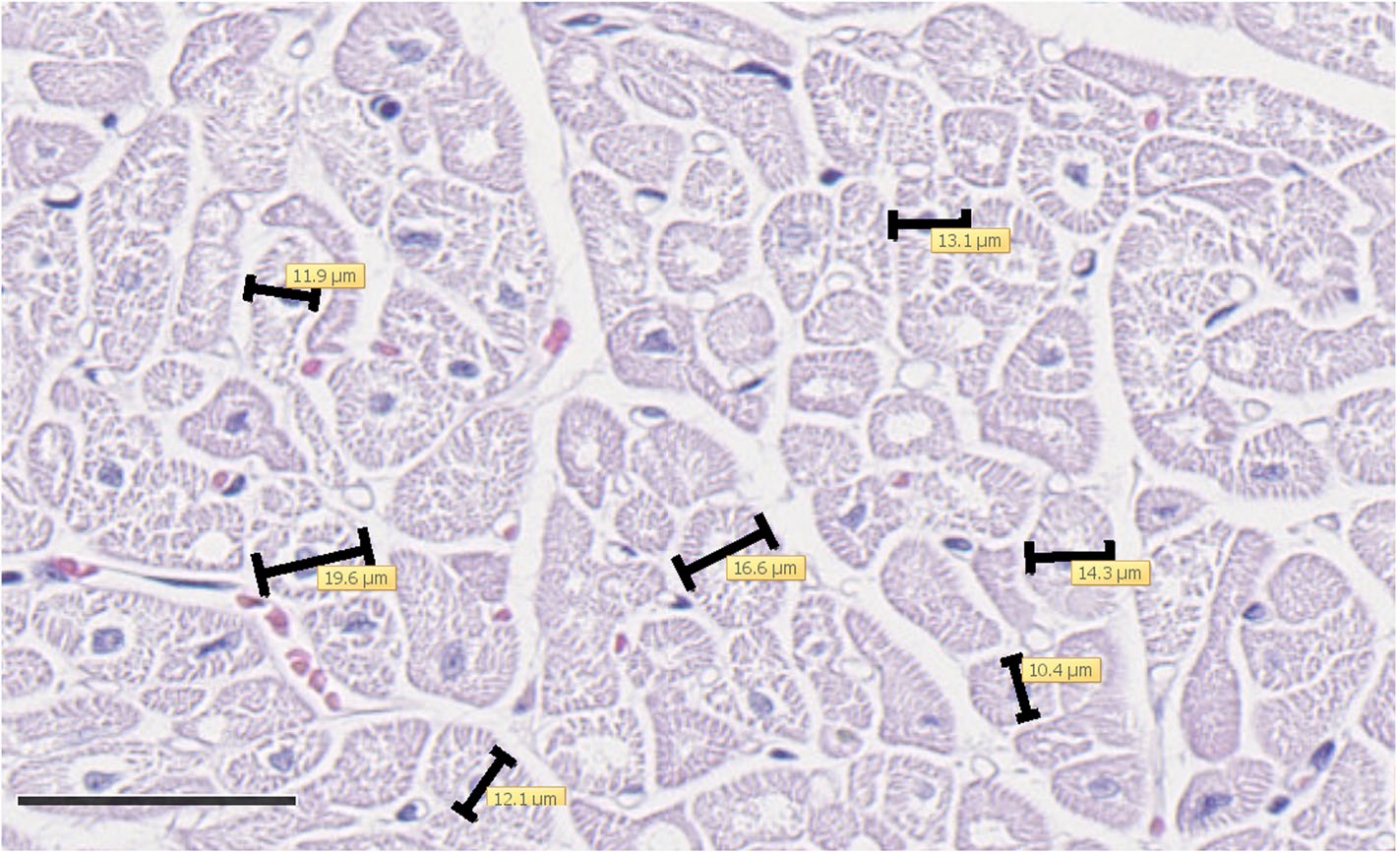
Example of cardiomyocyte diameter measurements. Measurement of the shortest diameter through the nucleus of cross-sectioned cardiomyocytes on hematoxylin and eosin stained sections (left corner bar is 50 μm). Example of FFC group animal

Cardiomyocytes with a central nucleus and cross-sectioned profile were selected, and the shortest diameter through the nucleus was measured on H&E stained sections using a software program (NDP.view2, Japan) (Fig. 2). An average diameter was calculated from 100 cardiomyocytes per section.

### Biochemical quantification of LV myocardial collagen content

Using a commercially available hydroxyproline assay kit, (Chondrex Inc., Redmond, WA, USA, CAT no.: 6017) LV collagen content was quantified on transmural LV snap-frozen samples. Before the assay, the LV samples were freeze-dried at − 55 °C (Heto FD3, Heto-Holten A/S, Allerød, Denmark) for 24 h, dry weight was determined and the freeze-dried samples were crushed using mortar and pestle. An acid hydrolysis was then performed using 200 μl 5 N hydrochloric acid per 10 mg wet weight cardiac tissue, and samples were incubated at 99 °C for 36 h according to Hanes and coworkers [34]. After incubation, samples were cooled to room temperature and centrifuged for 3 min at 9000×g. Supernatant was collected and the hydroxyproline assay was performed according to manufacturer’s instructions. All standards, samples and sample blanks were run in duplicate and rerun if coefficient of variance (CV) was more than 15%.

### Biochemical quantification of LV myocardial triglyceride content

LV transmural snap-frozen samples were kept cold on dry ice and crushed using mortar and pestle. Around 100 mg crushed LV tissue was mixed with 2 mL 0.15 M sodium acetate with triton-X100 (0.75%) to solubilize tissue lipids. Samples were then homogenized for 30 s with T25 digital Ultra Turrax® (IKA, Staufen, Germany) at 20000 repetitions per minute and immediately placed in a 90–100 °C water bath for 2 min and 15 s. After cooling down on wet ice, the samples were centrifuged at 3400×g for 10 min and supernatant was collected. Triglyceride analysis was performed using Cobas® 6000 auto analyzer according to manufacturer’s instructions and adjusted to tissue weight. All samples were run in duplicate and rerun if CV was more than 15%.

### Statistical analysis

Group differences between continuous variables were evaluated using a Kruskal-Wallis test with Wilcoxon Rank-sum post hoc test. ANOVA was not used due to lack of homogeneity of the residuals even after transformation. Fisher’s exact test was performed on categorical variables (epicardial fat, interstitial fat and interstitial fibrosis) to determine group differences.

To test for correlations between myocardial changes and body composition measurements (BW, body fat percentage, LBM), heart weight, circulating lipids (triglyceride, total cholesterol) and glucose metabolism markers (K_G_ and AUC_Insulin_) a Pearson correlation analysis was performed. A Shapiro-Wilk test was used to test for normality of response variable and if normality was not obtained, a non-parametrical Spearman correlation analysis was performed.

Data and results are presented as median and interquartile range (25th–75th). Statistical analyses were made using statistical software (SAS® 9.4, SAS Institute Inc., Cary, NC, USA). Graphical illustrations and calculation of K_G_ and AUC_Insulin_ were made using GraphPad Prism® (7.0c for Macintosh, GraphPad Software Inc., San Diego, CA, USA). A value of *P* < 0.05 was considered significant for the analyses.

## Results

Five out of forty animals did not complete the study. Four of these animals were euthanized before time; one due to lameness (FFC/SD *n* = 1); another due to spontaneous acute bleeding (FFC/SD *n* = 1) (diagnosed trombocytopenia purpura, a known background finding in Göttingen Minipigs [35]); and two (SD *n* = 1, FFC *n* = 1) as a result of procedure related complications. The last animal (FFC *n* = 1) died suddenly after a short period with acute respiratory distress, probably caused by circulatory failure.

### Body composition and heart weight

**Fig. 3:**
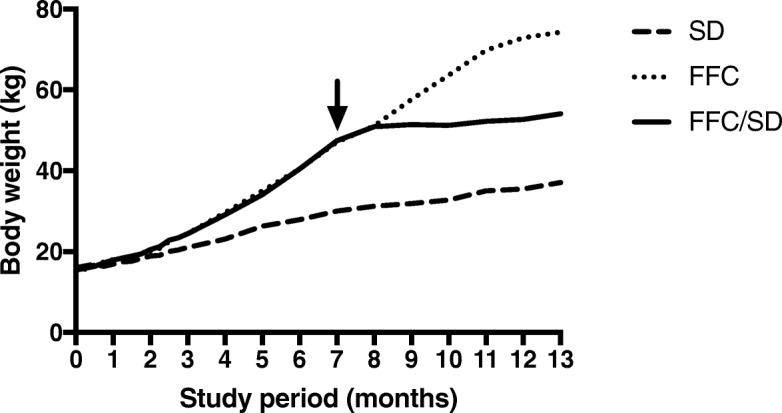
Body weight development throughout the study period for all three groups. All animals were weighed weekly. Arrow: Time point where FFC/SD animals were changed from a fat, fructose and cholesterol-rich diet (FFC) to standard minipig chow, which was approximately 8 months after study initiation. SD = fed standard chow, FFC/SD = dietary normalization

As depicted in Fig. 3, BW of FFC/SD stagnated compared to the remaining groups after dietary normalization. Body composition, absolute and relative heart weight data are presented in Table 1. Body weight, body fat percentage and LBM were significantly higher in FFC and FFC/SD compared to SD animals mid-study. No differences were observed between FFC and FFC/SD at this time point. At study termination BW and body fat percentage were significantly higher in FFC and FFC/SD compared to SD animals, and these body composition measurements were significantly lower in FFC/SD compared to FFC. No difference in LBM was observed between groups at the end of study.

**Table 1:** Body composition, absolute and relative heart weight

	SD *n* = 7	FFC *n* = 14	FFC/SD *n* = 14	Overall *P* value
BW (kg)				
Mid-study	31.0 (30.0–32.0)	50.0 (47.0–55.0)^***^	49.5 (47.0–54.0)^***^	0.0003
Termination	39.0 (38.0–41.0)	78.0 (69.0–81.0)^***^	55.0 (51.0–59.0)^***††††^	<.0001
Fat percentage (%)				
Mid-study	20.5 (18.5–26.3)	40.2 (37.6–41.9)^***^	41.6 (38.8–44.5)^***^	0.0001
Termination	27.6 (24.0–30.7)	64.2 (61.4–67.6)^***^	47.3 (41.2–50.2)^***††††^	<.0001
LBM (kg)				
Mid-study	22.8 (20.1–23.1)	27.1 (25.0–28.2)^**^	25.6 (24.4–26.3)^**^	0.002
Termination	26.9 (24.0–28.6)	27.0 (24.8–27.8)	29.5 (27.3–30.5)	0.2
HW (g)				
Termination	131 (115–142)	199 (181–208)^a***^	173 (168–180)^***^	<.0001
HW/BW (g/kg)				
Termination	3.3 (3.1–3.4)	2.6 (2.5–2.8)^a***^	3.3 (3.0–3.4)^††††^	<.0001
HW/LBM (g/kg)				
Termination	5.0 (4.2–5.2)	7.7 (6.8–8.2)^a***^	6.2 (5.9–6.7)^***†††^	<.0001

Data is presented as median and interquartile range (25th–75th)

*SD* fed standard chow, *FFC* fed fat/fructose/cholesterol-rich diet, *FFC/SD* dietary normalization, *BW* body weight, *LBM* lean body mass, *HW* heart weight

^**^P < 0.01; ^***^P < 0.001, significantly different from SD, ^†††^*P* < 0.001; ^††††^*P* < 0.0001, significantly different from FFC

^a^ *n* = 13

Heart weight was significantly higher in both FFC and FFC/SD compared to SD animals. Despite no statistical significant difference in heart weight between FFC and FFC/SD, a tendency towards a lower heart weight was noticed in FFC/SD (*P* = 0.06). Heart weight normalized for BW was significantly lower in FFC compared to both SD and FFC/SD. Furthermore, heart weight normalized for LBM was significantly higher in FFC and FFC/SD compared to SD animals and animals in FFC/SD had a significantly lower normalized heart weight compared to FFC.

### Circulating lipids and glucose metabolism markers

Circulating lipids and glucose metabolism markers are presented in Table 2. Mid-study, plasma triglyceride levels were significantly increased in FFC compared to SD animals, but no difference was observed between animals in FFC/SD and the two other groups. Animals in both FFC/SD and FFC had significantly increased total cholesterol levels compared to SD animals mid-study. At termination, plasma triglyceride and total cholesterol levels in FFC were significantly increased compared to animals in the two other groups. Furthermore, no differences in triglyceride or total cholesterol levels were observed between FFC/SD and SD animals at termination. Mid-study, K_G_ was significantly decreased in FFC and FFC/SD compared to SD animals suggesting altered glucose metabolism in the two groups. At termination, K_G_ in FFC was significantly decreased compared to the two other groups, whereas no difference between FFC/SD and SD was observed. No differences in fasting glucose levels or AUC_Insulin_ were observed between any of the groups at the two time points. The HPLC results of the pooled samples showed that the SD (HDL-C / LDL-C / VLDL-C: 48.06% / 50.70% / 1.24%) and FFC/SD (HDL-C / LDL-C / VLDL-C: 41.50% / 56.79% / 1.71%) groups had cholesterol fractions distributed predominantly in HDL and LDL, whereas the FFC group (HDL-C / LDL-C / VLDL-C: 13.12% / 47.74% / 39.14%) predominantly had cholesterol fractions distributed into LDL and VLDL. Though, data is not conclusive, a fat/fructose/cholesterol-rich diet feeding appeared to increase amount of LDL-C and VLDL-C, while diet normalization returned the cholesterol fractions to an almost normal distribution.

**Table 2:** Circulating lipids and glucose metabolism markers

	SD *n* = 7	FFC *n* = 14	FFC/SD *n* = 14	Overall *P* value
Triglyceride (mM)				
Mid-study	0.38 (0.29–0.41)	0.57 (0.37–0.76)^*^	0.48 (0.33–0.52)	0.04
Termination	0.34 (0.29–0.35)^b^	0.63 (0.54–0.88)^h**^	0.36 (0.32–0.45)^g†††^	<.0001
Total cholesterol (mM)				
Mid-study	2.0 (1.6–2.2)	14.1 (11.1–22.1)^***^	14.6 (11.2–18.7)^***^	0.0003
Termination	1.7 (1.6–2.2)^b^	11.9 (11.0–13.2)^h***^	1.9 (1.6–2.0)^g††††^	<.0001
Glucose (mM)				
Mid-study	3.8 (3.5–4.0)	4.0 (3.9–4.2)^h^	3.9 (3.6–4.2)^f^	0.3
Termination	3.4 (3.2–3.6)^b^	3.7 (3.6–3.9)	3.8 (3.7–4.0)^e^	0.05
K_G_ (min^−1^)				
Mid-study	3.1 (2.8–4.9)	2.2 (1.9–2.4)^h**^	2.1 (1.9–2.1)^**^	0.005
Termination	3.2 (2.7–3.5)^b^	2.1 (1.8–2.4)^h**^	3.2 (2.5–3.6)^†^	0.01
AUC_Insulin_ (pM*min)				
Mid-study	6983 (6344–12,602)	10,519 (9780–16,901)^h^	11,834 (10996–14,716)^d^	0.2
Termination	12,573 (11507–20,683)^a^	27,168 (17204–35,562)^h^	24,032 (20478–28,147)^c^	0.07

Data is presented as median and interquartile range (25th–75th)

*SD* fed standard chow, *FFC* fed fat/fructose/cholesterol-rich diet, *FFC/SD* dietary normalization, *K*_*G*_ intravenous glucose tolerance index, *AUC*_*Insulin*_ area under the curve of insulin

^*^P < 0.05; ^**^P < 0.01; ^***^P < 0.001, significantly different from SD, ^†^*P* < 0.05; ^†††^*P* < 0.001; ^††††^*P* < 0.0001, significantly different from FFC

^a^ *n* = 5

^b^ *n* = 6

^c^ *n* = 8

^d^ *n* = 9

^e^ *n* = 10

^f^ *n* = 11

^g^ *n* = 12

^h^ *n* = 13

### Left ventricular fibrosis

**Fig. 4:**
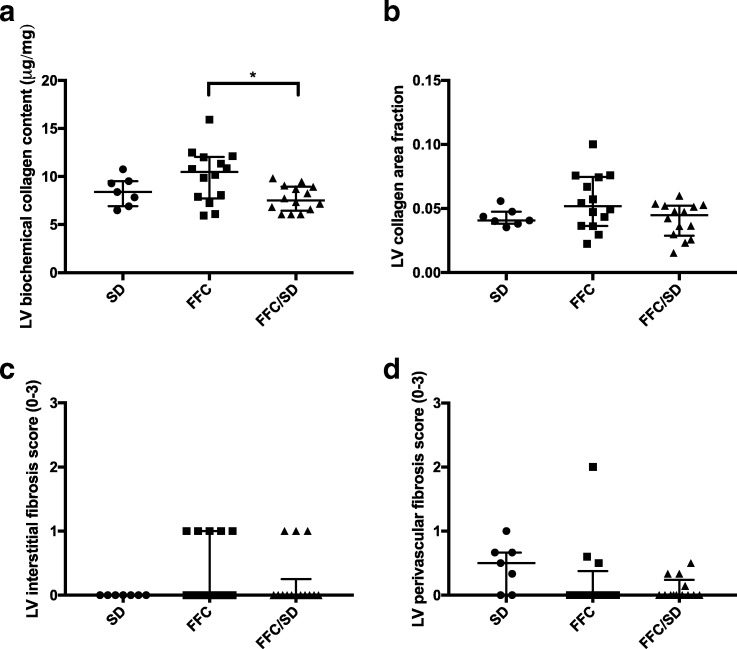
Left ventricular fibrosis. Left ventricular **a** collagen content quantified biochemically, **b** collagen area fraction assessed by digital image software and **c** interstitial and **d** perivascular fibrosis scores evaluated on histological sections stained with picro sirius red with Weigert’s iron hematoxylin nuclear staining. SD = fed standard chow, FFC = fed fat/fructose/cholesterol-rich diet, FFC/SD = dietary normalization. Black bars represent median and interquartile range (25th–75th). * = *P* < 0.05

Animals in FFC/SD had significantly lower LV myocardial collagen content compared to FFC when collagen content was assessed biochemically (*P* = 0.02). However, no difference between SD and FFC or FFC/SD was observed using this method (Fig. 4a). Quantification of collagen by image-analysis revealed no difference between groups (Fig. 4b), and further no significant difference in interstitial or perivascular fibrosis score was found between groups when evaluated semi-quantitative (Fig. 4c, d). However, a trend towards a difference in perivascular fibrosis between groups was noticed (*P* = 0.06, Fig. 4d). No signs of replacement fibrosis were found in any of the groups.

### Left ventricular fat

**Fig. 5:**
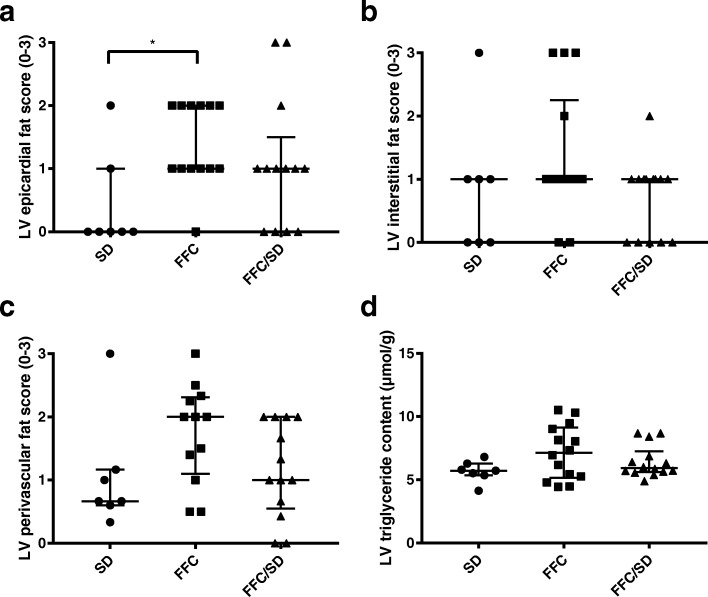
Left ventricular fat. Left ventricular **a** epicardial, **b** interstitial, **c** perivascular fat infiltration scores evaluated on hematoxylin and eosin stained sections and **d** left ventricular triglyceride content quantified biochemically. SD = fed standard chow, FFC = fed fat/fructose/cholesterol-rich diet, FFC/SD = dietary normalization. Black bars represent median and interquartile range (25th–75th). * = *P* < 0.05

Epicardial fat score differed between groups (*P* = 0.02). Higher scores were given to FFC animals compared to SD (*P* = 0.01), but no difference was found between FFC and FFC/SD, or FFC/SD and SD (Fig. 5a). However, no significant difference in interstitial or perivascular fat infiltration score was observed between groups (Fig. 5b, c). There was no difference in LV triglyceride content between groups when assessed biochemically (Fig. 5d).

### Cardiomyocyte hypertrophy

**Fig. 6:**
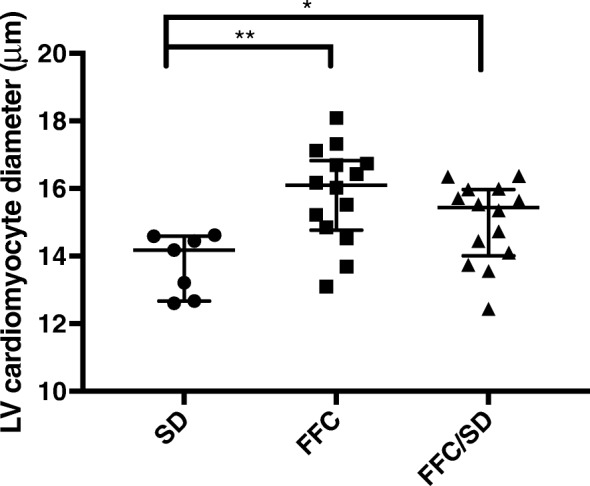
Left ventricular cardiomyocyte diameter. Left ventricular cardiomyocyte diameter assessed on hematoxylin and eosin stained sections. SD = fed standard chow, FFC = fed fat/fructose/cholesterol-rich diet, FFC/SD = dietary normalization. Black bars in graph represent median and interquartile range (25th–75th). * = *P* < 0.05, ** = *P* < 0.01

Cardiomyocyte diameter was significantly increased in FFC (*P* = 0.005) and FFC/SD (*P* = 0.04) compared to SD animals. Although not reaching statistical significance (*P* = 0.09), a trend towards lower cardiomyocyte diameter was observed in FFC/SD compared to FFC (Fig. 6).

### Correlation analyses

All correlation analyses can be seen in Table 3. Cardiomyocyte diameter was positively correlated with BW (*r* = 0.54, *P* = 0.0008), body fat percentage (*r* = 0.53, *P* = 0.001), and heart weight (*r* = 0.51, *P* = 0.002). LV triglyceride content assessed biochemically was also positively correlated with BW (r_s_ = 0.45, *P* = 0.007), body fat percentage (r_s_ = 0.35, *P* = 0.04), heart weight (r_s_ = 0.35, *P* = 0.04), and plasma triglyceride level (r_s_ = 0.37, *P* = 0.03). LV myocardial collagen content assessed biochemically tended to be positively correlated with body fat percentage (r_s_ = 0.32, *P* = 0.06), plasma total cholesterol level (r_s_ = 0.31, *P* = 0.08), plasma triglyceride level (r_s_ = 0.30, *P* = 0.08) and further LV myocardial collagen content tended to be inversely correlated with K_G_ (r_s_ = − 0.35, *P* = 0.06) and LBM (r_s_ = − 0.29, *P* = 0.09). Collagen level quantified by image-analysis tended to be positively correlated with BW (*r* = 0.29, *P* = 0.09), body fat percentage (*r* = 0.29, *P* = 0.09) and plasma total cholesterol level (*r* = 0.32, *P* = 0.07). Remaining correlations were not significant.

**Table 3:** Correlation analyses

	BW (kg)	Body fat (%)	LBM (kg)	HW (g)	Plasma TC (mM)	Plasma TG (mM)	AUC_Insulin_ (pmol/min)	K_G_ (min^−1^)
Biochemical collagen (μg/mg)	r_s_ = 0.25 *P* = 0.1	r_s_ = 0.32 *P* = 0.06	r_s_ = −0.29 *P* = 0.09	r_s_ = 0.015 *P* = 0.9	r_s_ = 0.31 *P* = 0.08	r_s_ = 0.30 *P* = 0.08	r_s_ = 0.13 *P* = 0.5	r_s_ = −0.35 *P* = 0.06
Collagen fraction area	*r* = 0.29 *P* = 0.09	*r* = 0.29 *P* = 0.09	*r* = −0.11 *P* = 0.5	*r* = 0.13 *P* = 0.5	*r* = 0.32 *P* = 0.07	*r* = 0.17 *P* = 0.3	*r* = 0.25 *P* = 0.2	*r* = −0.27 *P* = 0.2
Biochemical TG (μmol/g)	r_s_ = 0.45 ***P*** = 0.007**	r_s_ = 0.35 ***P*** = 0.04*	r_s_ = 0.18 *P* = 0.3	r_s_ = 0.35 ***P*** = 0.04*	r_s_ = 0.025 *P* = 0.9	r_s_ = 0.37 ***P*** = 0.03*	r_s_ = 0.12 *P* = 0.6	r_s_ = 0.006 *P* = 1.0
Cardiomyocyte diameter (μm)	*r* = 0.54 ***P*** = 0.0008***	*r* = 0.53 ***P*** = 0.001**	*r* = −0.046 *P* = 0.8	*r* = 0.51 ***P*** = 0.002**	*r* = 0.23 *P* = 0.2	*r* = 0.11 *P* = 0.5	*r* = 0.26 *P* = 0.2	*r* = −0.15 *P* = 0.4

*BW* body weight, *LBM* lean body mass, *HW* heart weight, *TC* total cholesterol, *TG* triglyceride, *AUC*_*Insulin*_ area under the curve of insulin, *K*_*G*_ intravenous glucose tolerance index; *P < 0.05; **P < 0.01; ***P < 0.001.

## Discussion

The main finding of the present study is that a dietary change from a fat/fructose/cholesterol-rich diet to standard minipig chow limited the amount of collagen in the myocardium of obese Göttingen Minipigs. However, dietary normalization did not have a significant beneficial effect on any of the other LV myocardial parameters investigated. In terms of other general health benefits of dietary normalization, BW gain was reduced, dyslipidemia was reversed and intravenous glucose tolerance index was improved.

Using a biochemical assessment of collagen, animals in FC/SD had significantly lower myocardial collagen content compared to FFC at termination. To date, no previous porcine studies investigating the effect of dietary normalization on reversal of myocardial pathological alteration have been reported. However, previous studies in obese rodents have explained the attenuation of myocardial fibrosis after a dietary intervention by a reduction in myocardial oxidative stress and inflammation [20, 21]. This is in line with porcine studies linking increased myocardial oxidative stress, inflammation and development of myocardial fibrosis [27, 30]. In a previous study of obese Göttingen Minipigs levels of oxidized low-density lipoprotein (oxLDL) and C-reactive protein, markers of systemic inflammation, were found to be increased after 22 weeks of a fat, fructose and cholesterol-rich diet [26]. Dietary fructose has been shown to contribute to inflammation in different tissues [36] and the dietary content of fructose in the present study may therefore have caused inflammation similar to the study by Ludvigsen and coworkers [26]. Furthermore, increased levels of oxLDL have been directly associated with stimulation of cardiac fibroblast signaling and collagen formation [37]. Regarding oxidative stress in the state of obesity, it has been proposed that hyperglycemia and insulin resistance may increase myocardial oxidative stress as a result of increased lipid metabolism [38]. In agreement with this, we found a tendency to an inverse correlation between LV myocardial collagen content and K_G_ in the present study. Moreover, a study by Kosmala and coworkers [39] showed that normal body mass index but increased body fat content was associated with a profibrotic state, indicating that adiposity may stimulate fibrosis formation. This is supported by the trend towards a positive correlation between LV collagen content and body fat percentage observed in the present study. A reduction in systemic oxidative stress and inflammatory markers was induced by 28-days of calorie restriction in overweight and obese women [40], suggesting that the timeframe of the present study might be sufficient to lower these parameters.

Studies of obese humans have shown that weight loss and maintenance of weight loss is important for improvements in LV geometry [16, 22], probably because the increase in body size is a driving factor for the hypertrophic pattern observed in obese subjects [41], due to pressure and volume overload associated with increasing body size [42]. In the present study, no significant difference in cardiomyocyte diameter or heart weight was observed between FFC and FFC/SD, even though BW was markedly lower in animals in FFC/SD. Autopsy studies of obese humans suggest increase of heart weight until a certain BW threshold, after which only modest heart weight changes are observed [9, 41, 43]. The lower heart weight/BW ratio observed in FFC compared to both SD and FFC/SD may suggest that such a threshold also exist in pigs where animals in FFC may have reached the BW threshold. This relation could also explain why the differences in heart weight and cardiomyocyte diameter between FFC and FC/SD were less pronounced and did not reach statistical significance in contrast body composition measurements. Heart weight was further normalized for LBM to quantify the degree of cardiac hypertrophy [9] and this revealed a significant difference between FFC and FFC/SD with animals in FFC having the largest ratio. Together with the heart weight/BW ratio, this may suggest that dietary normalization stopped the progression of cardiac hypertrophy. Correlation analyses also revealed positive correlations between obesity (BW and body fat percentage) and cardiomyocyte diameter, stating the importance of body size as a mediator of LV hypertrophy, also in the present study.

A positive correlation between cardiomyocyte diameter and heart weight was found in the current study, showing that cardiomyocyte hypertrophy is an important component of increase in cardiac weight. However, it is worth noticing that increased heart weight also can be caused by increased epicardial fat mass or myocardial fat infiltration [44, 45], which is supported by the positive correlation found between heart weight and LV triglyceride content. Furthermore, epicardial fat score assessed histologically was significantly higher in both FFC and FFC/SD compared to SD and this may also have contributed to the larger heart weight observed in these two groups.

A limitation of the present study was that no myocardial tissue was collected mid-study, prior to diet adjustment for FFC/SD and therefore it is difficult to determine if the dietary normalization reversed or just stopped the progression of the myocardial alterations. Tissue collection at this time and/or relevant in vivo assessments would therefore have been preferred. A transmural sample of LV was used for the biochemical assessment of collagen whereas the epi- and endocardium were not a part of the histological fibrosis analyses. An increase in epi- and/or endocardial collagen in FFC may therefore have influenced that a significant difference in collagen content only was observed when collagen was assessed biochemically. Compared to previous porcine studies [27, 30] only minor myocardial pathological alterations were induced in FFC despite 13 months of fat/fructose/cholesterol-rich diet. The reason might be difference between porcine breeds or differences in study setup e.g. castration. All animals were castrated before study initiation because castrated male Göttingen Minipigs become more prone to obesity and metabolic changes after castration [31]. Literature state that castration may protect against age-related myocardial fibrosis in mice [46], but also that castration promotes myocardial fibrosis formation in rodent heart failure models [47, 48]. However, to the authors’ knowledge, no studies have investigated this relationship in pigs. The lack of myocardial changes in the present study poses a limitation to the study and it could be speculated that more pronounced effects of dietary normalization would have been observed if more severe myocardial alterations had been induced.

## Conclusion and perspectives

Dietary change from a fat/fructose/cholesterol-rich diet to standard minipig chow limited the development of myocardial collagen content in obese Göttingen Minipigs compared to animals staying on the fat/fructose/cholesterol-rich diet for further 6 months. In addition, the degree of adiposity and glucose tolerance might have influenced myocardial collagen level. Plasma triglyceride and total cholesterol levels also improved after dietary normalization, however, plasma lipid levels did not correlate with myocardial collagen content. Only mild myocardial changes were found in this model of obesity and it is therefore important that future studies focus on inducing more severe myocardial changes before dietary or pharmacological interventions.

## Abbreviations

AUC_Insulin_: Insulin response during the intravenous glucose tolerance test
BW: Body weight
CV: Coefficient of variance
DEXA: Dual-energy x-ray absorptiometry
FFC: Obese animals fed fat/fructose/cholesterol-rich diet
FFC/SD: Diet normalization group
H&E: Hematoxylin and eosin
HF: Heart failure
IV: Intravenous
IVGTT: Intravenous glucose tolerance test
K_G_: Intravenous glucose tolerance index
LBM: Lean body mass
LV: Left ventricular
oxLDL: oxidized low-density lipoprotein
PSR: Picro sirius red
SD: Lean control animals fed standard minipig chow

## References

[1] World Health Organization. Obesity and overweight. http://www.who.int/mediacentre/factsheets/fs311/en/. 2018. Accessed 20 Mar 2018.

[2] Hubert HB, Feinleib M, McNamara PM, Castelli WP (1983). Obesity as an independent risk factor for cardiovascular disease: a 26-year follow-up of participants in the Framingham heart study. Circulation.

[3] Burke GL, Bertoni AG, Shea S, Tracy R, Watson KE, Blumenthal RS (2008). The impact of obesity on cardiovascular disease risk factors and subclinical vascular disease. Arch Intern Med.

[4] Kenchaiah S, Evans JC, Levy D, Wilson PWF, Benjamin EJ, Larson MG (2002). Obesity and the risk of heart failure. N Engl J Med.

[5] Gaborit B, Kober F, Jacquier a MPJ, Cuisset T, Boullu S (2012). Assessment of epicardial fat volume and myocardial triglyceride content in severely obese subjects: relationship to metabolic profile, cardiac function and visceral fat. Int J Obes.

[6] Kankaanpää M, Lehto H-R, Pärkkä JP, Komu M, Viljanen A, Ferrannini E (2006). Myocardial triglyceride content and epicardial fat mass in human obesity: relationship to left ventricular function and serum free fatty acid levels. J Clin Endocrinol Metab.

[7] Wende AR, Abel ED (1801). Lipotoxicity in the heart. Biochim Biophys Acta.

[8] Woodiwiss AJ, Libhaber CD, Majane OHI, Libhaber E, Maseko M, Norton GR (2008). Obesity promotes left ventricular concentric rather than eccentric geometric remodeling and hypertrophy independent of blood pressure. Am J Hypertens.

[9] Abel ED, Litwin SE, Sweeney G (2008). Cardiac remodeling in obesity. Physiol Rev.

[10] Rasmussen MH, Jensen LT, Andersen T, Breum L, Hilsted J (1995). Collagen metabolism in obesity: the effect of weight loss. Int J Obes Relat Metab Disord.

[11] Cavalera M, Wang J, Frangogiannis NG (2014). Obesity, metabolic dysfunction, and cardiac fibrosis: pathophysiological pathways, molecular mechanisms, and therapeutic opportunities. Transl Res.

[12] Shapiro LM, Gibson DG (1988). Patterns of diastolic dysfunction in left ventricular hypertrophy. Br Heart J.

[13] de Divitiis O, Fazio S, Petitto M, Maddalena G, Contaldo F, Mancini M (1981). Obesity and cardiac function. Circulation.

[14] Pascual M, D a P, Soria F, Vicente T, Hernández a M, Tébar FJ (2003). Effects of isolated obesity on systolic and diastolic left ventricular function. Heart.

[15] Russo C, Jin Z, Homma S, Rundek T, Elkind MSV, Sacco RL (2011). Effect of obesity and overweight on left ventricular diastolic function: a community-based study in an elderly cohort. J Am Coll Cardiol.

[16] de las Fuentes L, Waggoner AD, Mohammed BS, Stein RI, Miller BV, Foster GD (2009). Effect of moderate diet-induced weight loss and weight regain on cardiovascular structure and function. J Am Coll Cardiol.

[17] Viljanen APM, Karmi A, Borra R, Pärkkä JP, Lepomäki V, Parkkola R (2009). Effect of caloric restriction on myocardial fatty acid uptake, left ventricular mass, and cardiac work in obese adults. Am J Cardiol.

[18] Utz W, Engeli S, Haufe S, Kast P, Böhnke J, Haas V (2012). Moderate dietary weight loss reduces myocardial steatosis in obese and overweight women. J Cardiovasc Magn Reson.

[19] Haufe S, Utz W, Engeli S, Kast P, Böhnke J, Pofahl M (2012). Left ventricular mass and function with reduced-fat or reduced-carbohydrate hypocaloric diets in overweight and obese subjects. Hypertension.

[20] Takatsu M, Nakashima C, Takahashi K, Murase T, Hattori T, Ito H (2013). Calorie restriction attenuates cardiac remodeling and diastolic dysfunction in a rat model of metabolic syndrome. Hypertension.

[21] Wang H-T, Liu C-F, Tsai T-H, Chen Y-L, Chang H-W, Tsai C-Y (2012). Effect of obesity reduction on preservation of heart function and attenuation of left ventricular remodeling, oxidative stress and inflammation in obese mice. J Transl Med.

[22] Kosmala W, O’Moore-Sullivan T, Plaksej R, Przewlocka-Kosmala M, Marwick TH (2009). Improvement of left ventricular function by lifestyle intervention in obesity: contributions of weight loss and reduced insulin resistance. Diabetologia.

[23] Gutierrez K, Dicks N, Glanzner WG, Agellon LB, Bordignon V (2015). Efficacy of the porcine species in biomedical research. Front Genet.

[24] Swindle MM, Makin A, Herron AJ, Clubb FJ, Frazier KS (2012). Swine as models in biomedical research and toxicology testing. Vet Pathol.

[25] Lee J, Xu Y, Lu L, Bergman B, Leitner JW, Greyson C (2010). Multiple abnormalities of myocardial insulin signaling in a porcine model of diet-induced obesity. Am J Physiol Heart Circ Physiol.

[26] Ludvigsen TP, Kirk RK, Christoffersen BØ, Pedersen HD, Martinussen T, Kildegaard J (2015). Göttingen minipig model of diet-induced atherosclerosis: influence of mild streptozotocin-induced diabetes on lesion severity and markers of inflammation evaluated in obese, obese and diabetic, and lean control animals. J Transl Med.

[27] Mannheim D, Herrmann J, Bonetti PO, Lavi R, Lerman LO, Lerman A (2011). Simvastatin preserves diastolic function in experimental hypercholesterolemia independently of its lipid lowering effect. Atherosclerosis.

[28] Zhu XY, Daghini E, Rodriguez-Porcel M, Chade AR, Napoli C, Lerman A (2007). Redox-sensitive myocardial remodeling and dysfunction in swine diet-induced experimental hypercholesterolemia. Atherosclerosis.

[29] Xia J, Zhang Y, Xin L, Kong S, Chen Y, Yang S (2015). Global transcriptomic profiling of cardiac hypertrophy and fatty heart induced by long-term high-energy diet in bama miniature pigs. PLoS One.

[30] Li S-J, Liu C-H, Chu H-P, Mersmann HJ, Ding S-T, Chu C-H (2016). The high-fat diet induces myocardial fibrosis in the metabolically healthy obese minipigs - the role of ER stress and oxidative stress. Clin. Nutr.

[31] Christoffersen BO, Gade LP, Golozoubova V, Svendsen O, Raun K (2010). Influence of castration-induced testosterone and estradiol deficiency on obesity and glucose metabolism in male Göttingen minipigs. Steroids.

[32] Petersen SB, Lovmand JM, Honoré L, Jeppesen CB, Pridal L, Skyggebjerg O (2010). Comparison of a luminescent oxygen channeling immunoassay and an ELISA for detecting insulin aspart in human serum. J Pharm Biomed Anal.

[33] van Hoeven KH, Factor SM (1990). A comparison of the pathological spectrum of hypertensive, diabetic, and hypertensive-diabetic heart disease. Circulation.

[34] Hanes DW, Wong ML, Chang CWJ, Humphrey S, Grayson JK, Boyd WD (2015). Embolization of the first diagonal branch of the left anterior descending coronary artery as a porcine model of chronic trans-mural myocardial infarction. J Transl Med.

[35] Carrasco L, Madsen LW, Salguero FJ, Núñez A, Sánchez-Cordón P, Bollen P (2003). Immune complex-associated thrombocytopenic purpura syndrome in sexually mature Göttingen minipigs. J Comp Pathol.

[36] Veličković N, Djordjevic A, Vasiljević A, Bursać B, Milutinović DV, Matić G (2013). Tissue-specific regulation of inflammation by macrophage migration inhibitory factor and glucocorticoids in fructose-fed Wistar rats. Br J Nutr.

[37] Hu C, Dandapat A, Sun L, Khan JA, Liu Y, Hermonat PL (2008). Regulation of TGFβ1-mediated collagen formation by LOX-1: studies based on forced overexpression of TGFβ1in wild-type and LOX-1 knock-out mouse cardiac fibroblasts. J Biol Chem.

[38] Abel ED (2010). Free fatty acid oxidation in insulin resistance and obesity. Hear Metab.

[39] Kosmala W, Jedrzejuk D, Derzhko R, Przewlocka-Kosmala M, Mysiak A, Bednarek-Tupikowska G (2012). Left ventricular function impairment in patients with normal-weight obesity: contribution of abdominal fat deposition, profibrotic state, reduced insulin sensitivity, and proinflammatory activation. Circ Cardiovasc Imaging.

[40] Buchowski MS, Hongu N, Acra S, Wang L, Warolin J, Roberts LJ (2012). Effect of modest caloric restriction on oxidative stress in women, a randomized trial. PLoS One.

[41] Alpert MA, Omran J, Mehra A, Ardhanari S (2014). Impact of obesity and weight loss on cardiac performance and morphology in adults. Prog Cardiovasc Dis.

[42] Alexander JK, Dennis EW, Smith WG, Amad KH, Duncan CW, Austin RC (1962). Blood volume, cardiac output, and distribution of systemic blood flow in extreme obesity. Cardiovasc Res Cent Bull.

[43] Smith HL, Willius FA (1933). Adiposity of the heart. Arch Intern Med.

[44] Dorn GW, Robbins J, Sugden PH (2003). Phenotyping hypertrophy: eschew obfuscation. Circ Res.

[45] Murdolo G, Angeli F, Reboldi G, Di Giacomo L, Aita A, Bartolini C (2015). Left ventricular hypertrophy and obesity: only a matter of fat?. High Blood Press Cardiovasc Prev.

[46] Hewitson TD, Zhao C, Wigg B, Lee SW, Simpson ER, Boon WC, Samuel CS (2012). Relaxin and castration in male mice protect from, but testosterone exacerbates, age-related cardiac and renal fibrosis, whereas estrogens are an independent determinant of organ size. Endocrinology.

[47] Kang N, Fu L, Xu J, Han Y, Cao J, Sun J, Zheng M (2012). Testosterone improves cardiac function and alters angiotensin II receptors in isoproterenol-induced heart failure. Arch Cardiovasc Dis.

[48] Yang J, Wang F, Sun W, Dong Y, Li M, Fu L (2016). Testosterone replacement modulates cardiac metabolic remodeling after myocardial infarction by upregulating PPAR. PPAR Res.

